# Ectopic Thyroid at the Base of the Tongue of a Young Patient

**DOI:** 10.1155/2016/9174970

**Published:** 2016-09-26

**Authors:** Paulo Henrique de Souza Castro, Luiz Evaristo Ricci Volpato, Julia Tramujas, Alvaro Henrique Borges

**Affiliations:** ^1^Mato Grosso Cancer Hospital, Cuiabá, MT, Brazil; ^2^Master's Program in Integrated Dental Sciences of the University of Cuiabá, Cuiabá, MT, Brazil

## Abstract

Lingual thyroid is defined as an ectopic thyroid gland tissue located in the midline of the tongue base and it is uncommonly observed in clinical practice and is rare in children. This paper describes the surgical treatment of ectopic thyroid at the base of the tongue in a child. The chief complaint of the 12-year-old, melanodermic female patient was the difficulty to swallow for 15 days. The intraoral physical examination barely showed a nodular lesion at tongue base. The CT scan showed a round, well defined hyperdense lesion of approximately 25.8 mm at its largest diameter, with infiltrative growth in the posterior region of the base of the tongue. The proposed treatment was complete resection of the lesion. The histopathological diagnosis was lingual thyroid. After the diagnosis was established, the patient was referred to an endocrinologist for exams and medical follow-up. Lingual thyroid is a rare condition and its diagnosis in children is even rarer. Its approach should be transdisciplinary and should take into consideration the hormonal aspects of the patient in addition to the clinical condition of the lesion. In the presented case, the removal of the alteration was performed via conservative surgical procedure followed by immediate referral of the patient to the endocrinologist for a follow-up.

## 1. Introduction

Lingual thyroid is defined as an ectopic thyroid gland tissue located in the midline of the tongue base [1]. Lingual thyroid, which was first described in 1869 by Hickmann [2], is rarely observed in clinical practice [3].

Its incidence is estimated to be approximately 1 in every 100,000 people, with pronounced predominance of females of 4 cases : 1 to 7 : 1 [4]. Its occurrence is rare in children [5].

The tissue of the thyroid gland located ectopically at the base of the tongue can lead to symptoms such as dysphagia, dysphonia, upper airway obstruction, or bleeding and may be associated with thyroid dysfunction [6].

The aim of this study was to report a case of lingual thyroid in a 12-year-old girl.

## 2. Case Report

The 12-year-old, melanodermic female patient searched for treatment accompanied by her mother in the Mato Grosso Cancer Hospital's Department of Dentistry, complaining of difficulty to swallow for about 15 days.

An extraoral physical examination of the patient showed satisfactory mouth opening and free and palpable condyles, without changes.

**Figure 1:**
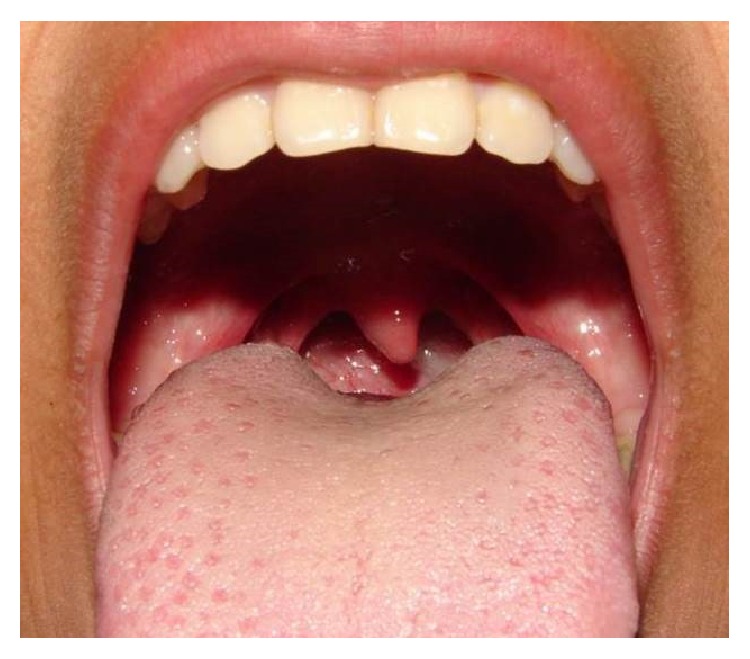
Intraoral physical examination showing a slight nodular lesion at the base of the tongue.

The intraoral physical examination showed asymptomatic nodular lesion at the base of the tongue, which was difficult to see (Figure 1).

**Figure 2:**
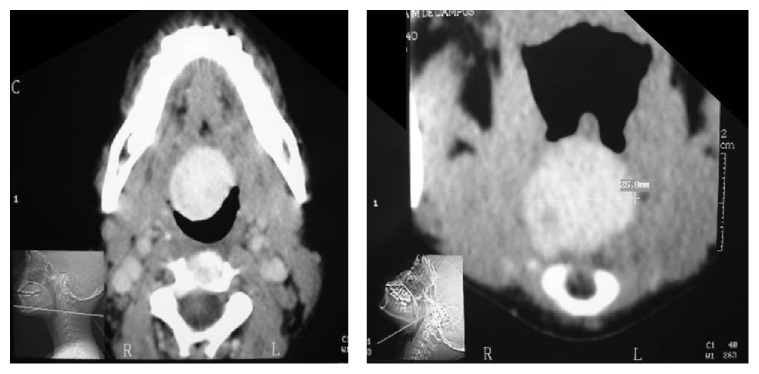
CT scan shows in axial cut a well defined hyperdense image of approximately 25.8 mm at its largest diameter, with infiltrative growth in the posterior region of the base of the tongue.

The CT scan showed a round, well defined hyperdense lesion of approximately 25.8 mm at its largest diameter, with infiltrative growth in the posterior region of the base of the tongue (Figure 2).

The proposed treatment was complete resection of the nodular lesion at the base of the tongue (excisional biopsy).

**Figure 3:**
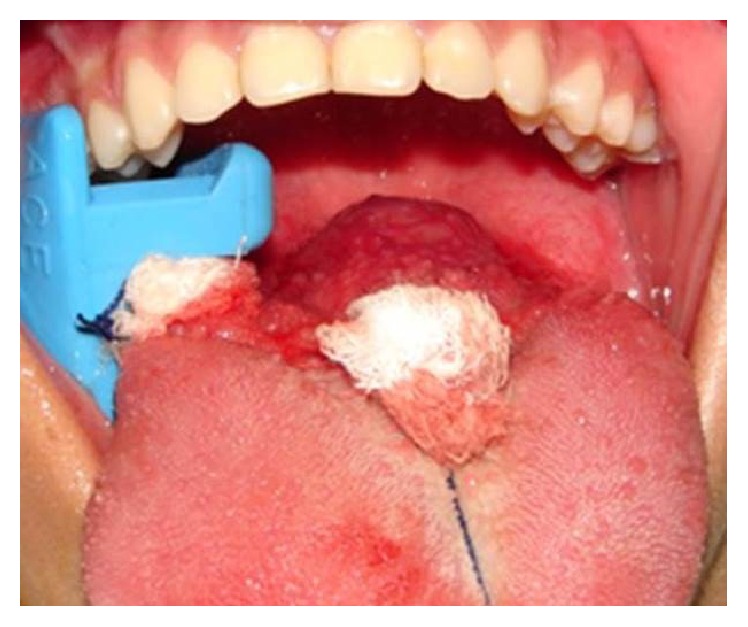
Nodular lesion at the base of the tongue after incision and detachment of the lingual mucosa.

**Figure 4 fig4:**
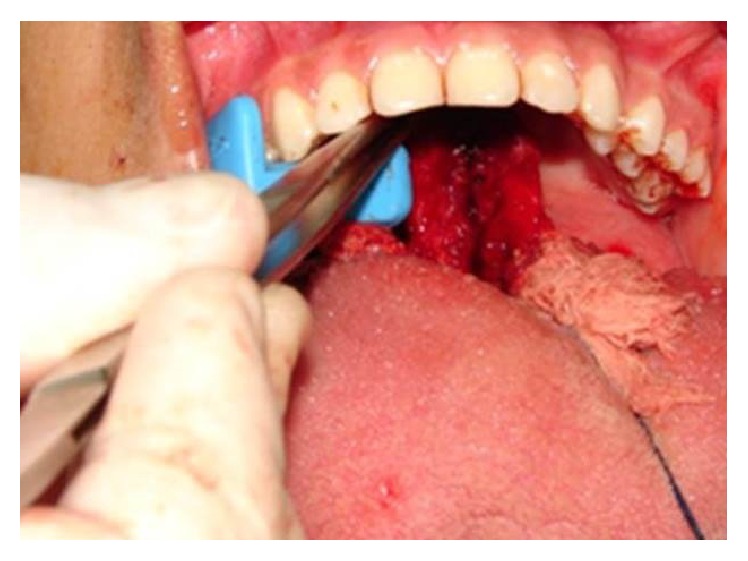
Excision of the lesion.

**Figure 5:**
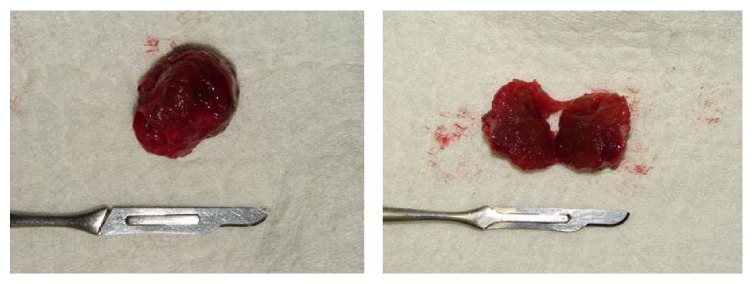
Red-colored and round-shaped nodular lesion.

The surgical procedure was performed using intraoral access under general anesthesia. After incision and detachment of the lingual mucosa, it was possible to visualize a red-colored round-shaped nodular lesion at the base of the tongue and to excise it (Figures 3 [Fig fig4]–5).

The patient remained hospitalized for twenty-four hours and then was discharged. The medications prescribed were cefalotin 1 g, dipyrone 1 g, ketoprofen 100 mg, hydrocortisone 500 mg, ranitidine 50 mg, and ondansetron 4 mg.

She reported improvement in swallowing after surgery, even though she was complaining of moderate pain in early postoperative period.

**Figure 6:**
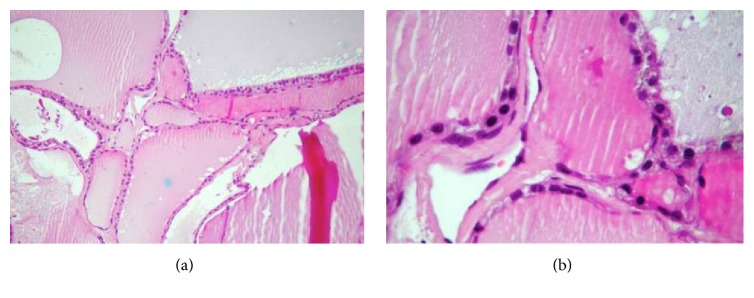
(a) The slide shows several follicular spaces with colloid content in their interior. (b) Colloid content surrounded by thyroid luminal epithelial cells classified as simple cuboidal glandular epithelium.

The histopathological diagnosis was lingual thyroid (Figure 6).

After the diagnosis of lingual thyroid was established, the patient was referred to an endocrinologist for exams and medical follow-up. The patient is currently under medical supervision, but there is no need for hormone replacement therapy.

The informed consent of the patient's legal guardian was obtained before submission of the manuscript.

## 3. Discussion

Embryologically, the thyroid develops from the floor of the primitive pharynx and migrates anteriorly and inferiorly until it reaches its final location in the adult [3] in the final pretracheal position [7]. The pathogenesis of the ectopia of the thyroid tissue is not clear; however, it has been postulated that maternal antithyroid antibodies would hinder the descent of the gland during embryogenesis [1]. Thus, the ectopic thyroid tissue is the result of abnormal embryonic development and migration of the gland. It may be observed anywhere along the downward path of the gland [7].

Most patients who have lingual thyroid show no symptoms. However, there are cases where the mass can be enlarged and cause dysphagia, dysphonia, dyspnea, or a feeling of suffocation [8]. In the reported case, the difficulty in swallowing was what triggered the search for diagnosis and treatment.

The diagnosis is mainly based on clinical and imaging examinations [5]. Palpation of the neck is essential in order to check for the presence or absence of the thyroid gland in its normal position. Thyroid function tests should also be performed, but this examination may often be normal [4]. The most important medical diagnostic tool is technetium TC-99m scanning with sodium, computed tomography, and magnetic resonance imaging [9].

In this case, it was possible to observe, through the CT scan, a round, well defined hyperdense nodular mass at the posterior region of the base of the tongue. An excisional biopsy of the lesion was performed and the piece of the lesion was sent for histopathological analysis for diagnostic purposes. At that moment, the possibility that the injury could be lingual thyroid had not yet been suggested.

Although in this case thyroid function and other imaging tests were not performed before the surgical intervention as, by then, the surgery team was not working with the possibility of lingual thyroid, it is imperative to emphasize their importance. The surgical excision of the lingual thyroid shall not be attempted until radioactive isotope scan has determined that there is adequate thyroid tissue in the neck.

The treatment for this alteration can be therapeutic or surgical and must take into account the physiological needs for thyroid hormones [5] and the severity of the symptoms [4]. The clinical control of lingual thyroid includes observation, suppressive therapy, and treatment with radioactive iodine [2].

The main complaint of the patient in this case was the difficulty in swallowing due to the volumetric increase in the base of the tongue, which is one of the indications for surgical removal of the lesion. Surgical intervention is indicated for symptomatic patients who present dyspnea, dysphagia, difficulty in speaking, or obstructive sleep apnea [2]. After histopathological diagnosis was confirmed in this case, the patient was referred to an endocrinologist for evaluation and conduct.

In most of these cases, a transcervical or transmandibular approach is required [2, 6]. In the present case, a more conservative surgical approach with intraoral access was possible, which provided a good postoperative outcome to the patient and avoided scars on the area of the neck.

## 4. Conclusion

Lingual thyroid is a rare condition and its diagnosis in children is even rarer. Its approach should be transdisciplinary and should take into consideration the hormonal aspects of the patient in addition to the clinical condition of the lesion. In the presented case, the removal of the alteration was performed via conservative surgical procedure followed by immediate referral of the patient to the endocrinologist for a follow-up.
